# Supplementary material for: ggpicrust2: an R package for PICRUSt2 predicted functional profile analysis and visualization
Source: Bioinformatics. 2023 Aug 1;39(8):btad470. doi: 10.1093/bioinformatics/btad470 (PMC10425198; doi:10.1093/bioinformatics/btad470)
Supplement: btad470_Supplementary_Data [file btad470_supplementary_data.zip › ggpicrust2 vigenett.pdf]

ggpicrust2 vigenett

Caffery Yang

2023-06-18

ggpicrust2 Documentation

ggpicrust2 is a comprehensive package designed to provide a seamless and intuitive solution for analyzing and interpreting the results of PICRUSt2 functional prediction. It offers a wide range of features, including pathway name/description annotations, advanced differential abundance (DA) methods, and visualization of DA results.

One of the newest additions to *ggpicrust2* is the capability to compare the consistency and inconsistency across different DA methods applied to the same dataset. This feature allows users to assess the agreement and discrepancy between various methods when it comes to predicting and sequencing the metagenome of a particular sample. It provides valuable insights into the consistency of results obtained from different approaches and helps users evaluate the robustness of their findings.

By leveraging this functionality, researchers, data scientists, and bioinformaticians can gain a deeper understanding of the underlying biological processes and mechanisms present in their PICRUSt2 output data. This comparison of different methods enables them to make informed decisions and draw reliable conclusions based on the consistency evaluation of macrogenomic predictions or sequencing results for the same sample.

If you are interested in exploring and analyzing your PICRUSt2 output data, *ggpicrust2* is a powerful tool that provides a comprehensive set of features, including the ability to assess the consistency and evaluate the performance of different methods applied to the same dataset.

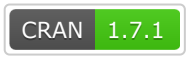

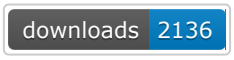

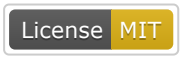

Table of Contents

- Citation
- Installation
- Workflow
- Function Details
  - ko2kegg_abundance()
  - pathway_daa()
  - compare_daa_results()
  - pathway_annotation()
  - pathway_errorbar()
  - pathway_heatmap()
  - pathway_pca()
  - compare_metagenome_results()
- FAQ

Citation

If you use *ggpicrust2* in your research, please cite the following paper:

Chen Yang, Aaron Burberry, Xuan Cao, Jiahao Mai, Liangliang Zhang. (2023). ggpicrust2: an R package for PICRUSt2 predicted functional profile analysis and visualization. arXiv preprint arXiv:2303.10388.

BibTeX entry: @misc{yang2023ggpicrust2, title={ggpicrust2: an R package for PICRUSt2 predicted functional profile analysis and visualization}, author={Chen Yang and Aaron Burberry and Jiahao Mai and Xuan Cao and Liangliang Zhang}, year={2023}, eprint={2303.10388}, archivePrefix={arXiv}, primaryClass={stat.AP} }

ResearchGate preprint link: [Click here]

Installation

You can install the stable version of *ggpicrust2* from CRAN with:

```
install.packages("ggpicrust2")
```

To install the latest development version of *ggpicrust2* from GitHub, you can use:

```
# Install the devtools package if not already installed
# install.packages("devtools")
# Install ggpicrust2 from GitHub
devtools::install_github("cafferychen777/ggpicrust2")
```

Dependent CRAN Packages

Package	Description
aplot	Create interactive plots
dplyr	A fast consistent tool for working with data frame like objects both in memory and out of memory
ggplot2	An implementation of the Grammar of Graphics in R
grid	A rewrite of the graphics layout capabilities of R
MicrobiomeStat	Statistical analysis of microbiome data
readr	Read rectangular data (csv tsv fwf) into R
stats	The R Stats Package
tibble	Simple Data Frames
tidyr	Easily tidy data with spread() and gather() functions
ggprism	Interactive 3D plots with ‘prism’ graphics
cowplot	Streamlined Plot Theme and Plot Annotations for ‘ggplot2’
ggforce	Easily add secondary axes, zooms, and image overlays to ‘ggplot2’
ggplotify	Convert complex plots into ‘grob’ or ‘ggplot’ objects
magrittr	A Forward-Pipe Operator for R
utils	The R Utils Package

Dependent Bioconductor Packages

Package	Description
phyloseq	Handling and analysis of high-throughput microbiome census data

Package	Description
ALDEx2	Differential abundance analysis of taxonomic and functional features
SummarizedExperiment	SummarizedExperiment container for storing data and metadata together
Biobase	Base functions for Bioconductor
devtools	Tools to make developing R packages easier
ComplexHeatmap	Making Complex Heatmaps in R
BiocGenerics	S4 generic functions for Bioconductor
BiocManager	Access the Bioconductor Project Package Repositories
metagenomeSeq	Statistical analysis for sparse high-throughput sequencing
Maaslin2	Tools for microbiome analysis
edgeR	Empirical Analysis of Digital Gene Expression Data in R
lefser	R implementation of the LEfSE method for microbiome biomarker discovery
limma	Linear Models for Microarray and RNA-Seq Data
KEGGREST	R Interface to KEGG REST API

```
if (!requireNamespace("BiocManager", quietly = TRUE))
        install.packages("BiocManager")
pkgs <- c("phyloseq", "ALDEx2", "SummarizedExperiment", "Biobase", "devtools",
          "ComplexHeatmap", "BiocGenerics", "BiocManager", "metagenomeSeq",
          "Maaslin2", "edgeR", "lefser", "limma", "KEGGREST")
for (pkg in pkgs) {
  if (!requireNamespace(pkg, quietly = TRUE))
    BiocManager::install(pkg)
}
```

Workflow

The easiest way to analyze the PICRUSt2 output is using ggpicrust2() function. The main pipeline can be run with ggpicrust2() function.

ggpicrust2() integrates ko abundance to kegg pathway abundance conversion, annotation of pathway, differential abundance (DA) analysis, part of DA results visualization. When you have trouble running ggpicrust2(), you can debug it by running a separate function, which will greatly increase the speed of your analysis and visualization.

ggpicrust2()

You can download the example dataset from the provided Github link and Google Drive link or use the dataset included in the package.

```
# If you want to analyze the abundance of KEGG pathways instead of KO within the pathway, please
        set `ko_to_kegg` to TRUE.
# KEGG pathways typically have more descriptive explanations.
library(readr)
library(ggpicrust2)
library(tibble)
```

```
library(tidyverse)
library(ggprism)
library(patchwork)
# Load necessary data: abundance data and metadata
abundance_file <- "path/to/your/abundance_file.tsv"
metadata <- read_delim(
    "path/to/your/metadata.txt",
    delim = "\t",
    escape_double = FALSE,
    trim_ws = TRUE
)

# Run ggpicrust2 with input file path
results_file_input <- ggpicrust2(file = abundance_file,
                                 metadata = metadata,
                                 group = "your_group_column", # For example dataset, group =
        "Environment"
                                 pathway = "KO",
                                 daa_method = "LinDA",
                                 ko_to_kegg = TRUE,
                                 order = "pathway_class",
                                 p_values_bar = TRUE,
                                 x_lab = "pathway_name")
# Run ggpicrust2 with imported data.frame
abundance_data <- read_delim(abundance_file, delim = "\t", col_names = TRUE, trim_ws = TRUE)
# Run ggpicrust2 with input data
results_data_input <- ggpicrust2(data = abundance_data,
                                 metadata = metadata,
                                 group = "your_group_column", # For example dataset, group =
        "Environment"
                                 pathway = "KO",
                                 daa_method = "LinDA",
                                 ko_to_kegg = TRUE,
                                 order = "pathway_class",
                                 p_values_bar = TRUE,
                                 x_lab = "pathway_name")
# Access the plot and results dataframe for the first DA method
example_plot <- results_file_input[[1]]$plot
example_results <- results_file_input[[1]]$results
# Use the example data in ggpicrust2 package
data(ko_abundance)
data(metadata)
results_file_input <- ggpicrust2(data = ko_abundance,
                                 metadata = metadata,
                                 group = "Environment",
                                 pathway = "KO",
                                 daa_method = "LinDA",
                                 ko_to_kegg = TRUE,
                                 order = "pathway_class",
                                 p_values_bar = TRUE,
                                 x_lab = "pathway_name")
# Analyze the EC or MetaCyc pathway
data(metacyc_abundance)
results_file_input <- ggpicrust2(data = metacyc_abundance,
                                 metadata = metadata,
                                 group = "Environment",
                                 pathway = "MetaCyc",
                                 daa_method = "LinDA",
                                 ko_to_kegg = FALSE,
                                 order = "group",
                                 p_values_bar = TRUE,
                                 x_lab = "description")
results_file_input[[1]]$plot
results_file_input[[1]]$results
```

If an error occurs with ggpicrust2, please use the following workflow.

```
library(readr)
library(ggpicrust2)
library(tibble)
library(tidyverse)
library(ggprism)
library(patchwork)
# If you want to analyze KEGG pathway abundance instead of KO within the pathway, turn ko_to_kegg
    to TRUE.
# KEGG pathways typically have more explainable descriptions.
# Load metadata as a tibble
# data(metadata)
metadata <- read_delim("path/to/your/metadata.txt", delim = "\t", escape_double = FALSE, trim_ws =
    TRUE)
# Load KEGG pathway abundance
# data(kegg_abundance)
kegg_abundance <- ko2kegg_abundance("path/to/your/pred_metagenome_unstrat.tsv")
# Perform pathway differential abundance analysis (DAA) using ALDEx2 method
# Please change group to "your_group_column" if you are not using example dataset
daa_results_df <- pathway_daa(abundance = kegg_abundance, metadata = metadata, group =
    "Environment", daa_method = "ALDEx2", select = NULL, reference = NULL)
# Filter results for ALDEx2_Welch's t test method
# Please check the unique(daa_results_df$method) and choose one
daa_sub_method_results_df <- daa_results_df[daa_results_df$method == "ALDEx2_Wilcoxon rank test",
    ]
# Annotate pathway results using KO to KEGG conversion
daa_annotated_sub_method_results_df <- pathway_annotation(pathway = "KO", daa_results_df =
    daa_sub_method_results_df, ko_to_kegg = TRUE)
# Generate pathway error bar plot
# Please change Group to metadata$your_group_column if you are not using example dataset
p <- pathway_errorbar(abundance = kegg_abundance, daa_results_df =
    daa_annotated_sub_method_results_df, Group = metadata$Environment, p_values_threshold =
    0.05, order = "pathway_class", select = NULL, ko_to_kegg = TRUE, p_value_bar = TRUE,
    colors = NULL, x_lab = "pathway_name")
# If you want to analyze EC, MetaCyc, and KO without conversions, turn ko_to_kegg to FALSE.
# Load metadata as a tibble
# data(metadata)
metadata <- read_delim("path/to/your/metadata.txt", delim = "\t", escape_double = FALSE, trim_ws =
    TRUE)
# Load KO abundance as a data.frame
# data(ko_abundance)
ko_abundance <- read.delim("path/to/your/pred_metagenome_unstrat.tsv")
# Perform pathway DAA using ALDEx2 method
# Please change column_to_rownames() to the feature column if you are not using example dataset
# Please change group to "your_group_column" if you are not using example dataset
daa_results_df <- pathway_daa(abundance = ko_abundance %>% column_to_rownames("#NAME"), metadata =
    metadata, group = "Environment", daa_method = "ALDEx2", select = NULL, reference = NULL)
# Filter results for ALDEx2_Kruskal-Wallace test method
daa_sub_method_results_df <- daa_results_df[daa_results_df$method == "ALDEx2_Wilcoxon rank test",
    ]
# Annotate pathway results without KO to KEGG conversion
daa_annotated_sub_method_results_df <- pathway_annotation(pathway = "KO", daa_results_df =
    daa_sub_method_results_df, ko_to_kegg = FALSE)
# Generate pathway error bar plot
# Please change column_to_rownames() to the feature column
# Please change Group to metadata$your_group_column if you are not using example dataset
p <- pathway_errorbar(abundance = ko_abundance %>% column_to_rownames("#NAME"), daa_results_df =
    daa_annotated_sub_method_results_df, Group = metadata$Environment, p_values_threshold =
    0.05, order = "group",
select = daa_annotated_sub_method_results_df %>% arrange(p_adjust) %>% slice(1:20) %>%
    dplyr::select(feature) %>% pull(),
ko_to_kegg = FALSE,
```

```
  p_value_bar = TRUE,
  colors = NULL,
  [illegible]"description")
  [illegible]ow for MetaCyc Pathway and EC
  # Load MetaCyc pathway abundance and metadata
  data("metacyc_abundance")
  [illegible]tadata")
  # Perform pathway DAA using LinDA method
  # Please change column_to_rownames() to the feature column if you are not using example dataset
  # Please change group to "your_group_column" if you are not using example dataset
  metacyc_daa_results_df <- pathway_daa(abundance = metacyc_abundance %>%
          column_to_rownames("pathway"), metadata = metadata, group = "Environment", daa_method =
          "LinDA")
  # Annotate MetaCyc pathway results without KO to KEGG conversion
  metacyc_daa_annotated_results_df <- pathway_annotation(pathway = "MetaCyc", daa_results_df =
          daa_results_df, ko_to_kegg = FALSE)
  # Generate pathway error bar plot
  # Please change column_to_rownames() to the feature column
  # Please change Group to metadata$your_group_column if you are not using example dataset
  pathway_errorbar(abundance = metacyc_abundance %>% column_to_rownames("pathway"), daa_results_df =
          metacyc_daa_annotated_results_df, Group = metadata$Environment, ko_to_kegg = FALSE,
          p_values_threshold = 0.05, order = "group", select = NULL, p_value_bar = TRUE, colors =
          NULL, x_lab = "description")
  # Generate pathway heatmap
  # Please change column_to_rownames() to the feature column if you are not using example dataset
  # Please change group to "your_group_column" if you are not using example dataset
  feature_with_p_0.05 <- metacyc_daa_results_df %>% filter(p_adjust < 0.05)
  pathway_heatmap(abundance = metacyc_abundance %>% filter(pathway %in% feature_with_p_0.05$feature)
          %>% column_to_rownames("pathway"), metadata = metadata, group = "Environment")
  # Generate pathway PCA plot
  # Please change column_to_rownames() to the feature column if you are not using example dataset
  # Please change group to "your_group_column" if you are not using example dataset
  pathway_pca(abundance = metacyc_abundance %>% column_to_rownames("pathway"), metadata = metadata,
          group = "Environment")
  # Run pathway DAA for multiple methods
  # Please change column_to_rownames() to the feature column if you are not using example dataset
  # Please change group to "your_group_column" if you are not using example dataset
  methods <- c("ALDEx2", "DESeq2", "edgeR")
  daa_results_list <- lapply(methods, function(method) {
    pathway_daa(abundance = metacyc_abundance %>% column_to_rownames("pathway"), metadata =
          metadata, group = "Environment", daa_method = method)
  [illegible]
  # Compare results across different methods
  comparison_results <- compare_daa_results(daa_results_list = daa_results_list, method_names =
          c("ALDEx2_Welch's t test", "ALDEx2_Wilcoxon rank test", "DESeq2", "edgeR"))
```

Output

The typical output of the ggpicrust2 is like this. This image is omitted due to the CRAN package size check issue.

function details

ko2kegg_abundance()

KEGG Orthology(KO) is a classification system developed by the Kyoto Encyclopedia of Genes and Genomes (KEGG) data-base(Kanehisa et al., 2022). It uses a hierarchical structure to classify enzymes based on the reactions they catalyze. To better understand pathways' role in different groups and classify the pathways, the KO abundance table needs to be converted to KEGG pathway abundance. But PICRUSt2 removes the function from PICRUSt. ko2kegg_abundance() can help convert the table.

```
# usage of the ko2kegg_abundance function
library(ggpicrust2)
# Assume that the KO abundance table is stored in a file named "ko_abundance.tsv"
ko_abundance_file <- "ko_abundance.tsv"
# Convert KO abundance to KEGG pathway abundance
kegg_abundance <- ko2kegg_abundance(file = ko_abundance_file)
# Alternatively, if the KO abundance data is already loaded as a data frame named "ko_abundance"
data("ko_abundance")
kegg_abundance <- ko2kegg_abundance(data = ko_abundance)
# The resulting kegg_abundance data frame can now be used for further analysis and visualization.
```

pathway_daa()

Differential abundance(DA) analysis plays a major role in PICRUSt2 downstream analysis. pathway_daa() integrates almost all DA methods applicable to the predicted functional profile which there excludes ANCOM and ANCOMBC. It includes ALDEx2(Fernandes et al., 2013), DESeq2(Love et al., 2014), Maaslin2(Mallick et al., 2021), LinDA(Zhou et al., 2022), edgeR(Robinson et al., 2010) , limma voom(Ritchie et al., 2015), metagenomeSeq(Paulson et al., 2013), Lefser(Segata et al., 2011).

```
# The abundance table is recommended to be a data.frame rather than a tibble.
# The abundance table should have feature names or pathway names as row names, and sample names as
    column names.
# You can use the output of ko2kegg_abundance
ko_abundance_file <- "path/to/your/pred_metagenome_unstrat.tsv"
kegg_abundance <- ko2kegg_abundance(ko_abundance_file) # Or use data(kegg_abundance)
metadata <- read_delim("path/to/your/metadata.txt", delim = "\t", escape_double = FALSE, trim_ws =
    TRUE)
# The default DAA method is "ALDEx2"
# Please change group to "your_group_column" if you are not using example dataset
daa_results_df <- pathway_daa(abundance = kegg_abundance, metadata = metadata, group =
    "Environment", daa_method = "linDA", select = NULL, p.adjust = "BH", reference = NULL)
# If you have more than 3 group levels and want to use the LinDA, limma voom, or Maaslin2 methods,
    you should provide a reference.
metadata <- read_delim("path/to/your/metadata.txt", delim = "\t", escape_double = FALSE, trim_ws =
    TRUE)
# Please change group to "your_group_column" if you are not using example dataset
daa_results_df <- pathway_daa(abundance = kegg_abundance, metadata = metadata, group = "Group",
    daa_method = "LinDA", select = NULL, p.adjust = "BH", reference = "Harvard BRI")
# Other example
data("metacyc_abundance")
data("metadata")
metacyc_daa_results_df <- pathway_daa(abundance = metacyc_abundance %>%
    column_to_rownames("pathway"), metadata = metadata, group = "Environment", daa_method =
    "LinDA", select = NULL, p.adjust = "BH", reference = NULL)
```

compare_daa_results()

```
library(ggpicrust2)
library(tidyverse)
data("metacyc_abundance")
data("metadata")
# Run pathway_daa function for multiple methods
# Please change column_to_rownames() to the feature column if you are not using example dataset
# Please change group to "your_group_column" if you are not using example dataset
methods <- c("ALDEx2", "DESeq2", "edgeR")
daa_results_list <- lapply(methods, function(method) {
  pathway_daa(abundance = metacyc_abundance %>% column_to_rownames("pathway"), metadata =
    metadata, group = "Environment", daa_method = method)
```

```
method_names <- c("ALDEx2_Welch's t test","ALDEx2_Wilcoxon rank test","DESeq2", "edgeR")
# Compare results across different methods
comparison_results <- compare_daa_results(daa_results_list = daa_results_list, method_names =
        method_names)
```

pathway_annotation()

If you are in China and you are using kegg pathway annotation, Please make sure your internet can break through the firewall.

```
    ure to check if the features in `daa_results_df` correspond to the selected pathway
# Annotate KEGG Pathway
data("kegg_abundance")
data("metadata")
# Please change group to "your_group_column" if you are not using example dataset
daa_results_df <- pathway_daa(abundance = kegg_abundance, metadata = metadata, group =
        "Environment", daa_method = "LinDA")
daa_annotated_results_df <- pathway_annotation(pathway = "KO", daa_results_df = daa_results_df,
        ko_to_kegg = TRUE)
# Annotate KO
data("ko_abundance")
data("metadata")
# Please change column_to_rownames() to the feature column if you are not using example dataset
# Please change group to "your_group_column" if you are not using example dataset
daa_results_df <- pathway_daa(abundance = ko_abundance %>% column_to_rownames("#NAME"), metadata =
        metadata, group = "Environment", daa_method = "LinDA")
daa_annotated_results_df <- pathway_annotation(pathway = "KO", daa_results_df = daa_results_df,
        ko_to_kegg = FALSE)
# Annotate KEGG
# daa_annotated_results_df <- pathway_annotation(pathway = "EC", daa_results_df = daa_results_df,
        ko_to_kegg = FALSE)
# Annotate MetaCyc Pathway
data("metacyc_abundance")
data("metadata")
# Please change column_to_rownames() to the feature column if you are not using example dataset
# Please change group to "your_group_column" if you are not using example dataset
metacyc_daa_results_df <- pathway_daa(abundance = metacyc_abundance %>%
        column_to_rownames("pathway"), metadata = metadata, group = "Environment", daa_method =
        "LinDA")
metacyc_daa_annotated_results_df <- pathway_annotation(pathway = "MetaCyc", daa_results_df =
        metacyc_daa_results_df, ko_to_kegg = FALSE)
```

pathway_errorbar()

```
data("ko_abundance")
data("metadata")
kegg_abundance <- ko2kegg_abundance(data = ko_abundance) # Or use data(kegg_abundance)
# Please change group to "your_group_column" if you are not using example dataset
daa_results_df <- pathway_daa(kegg_abundance, metadata = metadata, group = "Environment",
        daa_method = "LinDA")
daa_annotated_results_df <- pathway_annotation(pathway = "KO", daa_results_df = daa_results_df,
        ko_to_kegg = TRUE)
# Please change Group to metadata$your_group_column if you are not using example dataset
p <- pathway_errorbar(abundance = kegg_abundance,
           daa_results_df = daa_annotated_results_df,
           Group = metadata$Environment,
           ko_to_kegg = TRUE,
           p_values_threshold = 0.05,
           order = "pathway_class",
           select = NULL,
```

```
            p_value_bar = TRUE,
            colors = NULL,
            x_lab = "pathway_name")
# If you want to analysis the EC. MetaCyc. KO without conversions.
data("metacyc_abundance")
data("metadata")
metacyc_daa_results_df <- pathway_daa(abundance = metacyc_abundance %>%
        column_to_rownames("pathway"), metadata = metadata, group = "Environment", daa_method =
        "LinDA")
metacyc_daa_annotated_results_df <- pathway_annotation(pathway = "MetaCyc", daa_results_df =
        metacyc_daa_results_df, ko_to_kegg = FALSE)
p <- pathway_errorbar(abundance = metacyc_abundance %>% column_to_rownames("pathway"),
            daa_results_df = metacyc_daa_annotated_results_df,
            Group = metadata$Environment,
            ko_to_kegg = FALSE,
            p_values_threshold = 0.05,
            order = "group",
            select = NULL,
            p_value_bar = TRUE,
            colors = NULL,
            x_lab = "description")
```

pathway_heatmap()

In this section, we will demonstrate how to create a pathway heatmap using the `pathway_heatmap` function in the ggpicrust2 package. This function visualizes the relative abundance of pathways in different samples.

Use the fake dataset

```
# Create example functional pathway abundance data
abundance_example <- matrix(rnorm(30), nrow = 3, ncol = 10)
colnames(abundance_example) <- paste0("Sample", 1:10)
        (abundance_example) <- c("PathwayA", "PathwayB", "PathwayC")
# Create example metadata
# Please change your sample id's column name to sample_name
metadata_example <- data.frame(sample_name = colnames(abundance_example),
                               group = factor(rep(c("Control", "Treatment"), each = 5)))
# Create a heatmap
pathway_heatmap(abundance_example, metadata_example, "group")
```

Use the real dataset

```
# Load the data
        tacyc_abundance")
# Load the metadata
        tadata")
# Perform differential abundance analysis
metacyc_daa_results_df <- pathway_daa(
  abundance = metacyc_abundance %>% column_to_rownames("pathway"),
  metadata = metadata,
  group = "Environment",
  daa_method = "LinDA"
)
# Annotate the results
annotated_metacyc_daa_results_df <- pathway_annotation(
  pathway = "MetaCyc",
  daa_results_df = metacyc_daa_results_df,
  ko_to_kegg = FALSE
)
# Filter features with p < 0.05
feature_with_p_0.05 <- metacyc_daa_results_df %>%
        (p_adjust < 0.05)
```

```
# Create the heatmap
pathway_heatmap(
  abundance = metacyc_abundance %>%
    right_join(
      annotated_metacyc_daa_results_df %>% select(all_of(c("feature","description"))),
      by = c("pathway" = "feature")
    ) %>%
    filter(pathway %in% feature_with_p_0.05$feature) %>%
    select(-"pathway") %>%
    column_to_rownames("description"),
  metadata = metadata,
  group = "Environment"
)
```

pathway_pca()

In this section, we will demonstrate how to perform Principal Component Analysis (PCA) on functional pathway abundance data and create visualizations of the PCA results using the `pathway_pca` function in the ggpicrust2 package.

Use the fake dataset

```
# Create example functional pathway abundance data
abundance_example <- matrix(rnorm(30), nrow = 3, ncol = 10)
colnames(abundance_example) <- paste0("Sample", 1:10)
        (kegg_abundance_example) <- c("PathwayA", "PathwayB", "PathwayC")
# Create example metadata
metadata_example <- data.frame(sample_name = colnames(kegg_abundance_example),
                               group = factor(rep(c("Control", "Treatment"), each = 5)))
# Perform PCA and create visualizations
pathway_pca(abundance = abundance_example, metadata = metadata_example, "group")
```

Use the real dataset

```
# Create example functional pathway abundance data
data("metacyc_abundance")
        tadata")
pathway_pca(abundance = metacyc_abundance %>% column_to_rownames("pathway"), metadata = metadata,
        group = "Environment")
```

compare_metagenome_results()

```
library(ComplexHeatmap)
set.seed(123)
# First metagenome
metagenome1 <- abs(matrix(rnorm(1000), nrow = 100, ncol = 10))
rownames(metagenome1) <- paste0("KO", 1:100)
colnames(metagenome1) <- paste0("sample", 1:10)
# Second metagenome
metagenome2 <- abs(matrix(rnorm(1000), nrow = 100, ncol = 10))
rownames(metagenome2) <- paste0("KO", 1:100)
colnames(metagenome2) <- paste0("sample", 1:10)
# Put the metagenomes into a list
metagenomes <- list(metagenome1, metagenome2)
# Define names
names <- c("metagenome1", "metagenome2")
# Call the function
results <- compare_metagenome_results(metagenomes, names)
# Print the correlation matrix
```

```
print(results$correlation$cor_matrix)
# Print the p-value matrix
print(results$correlation$p_matrix)
```

FAQ

Issue 1: pathway_errorbar error

When using `pathway_errorbar` with the following parameters:

```
pathway_errorbar(abundance = abundance,
                 daa_results_df = daa_results_df,
                 Group = metadata$Environment,
                 ko_to_kegg = TRUE,
                 p_values_threshold = 0.05,
                 order = "pathway_class",
                 select = NULL,
                 p_value_bar = TRUE,
                 colors = NULL,
                 x_lab = "pathway_name")
```

You may encounter an error:

```
Error in `ggplot_add()`:
! Can't add `e2` to a <ggplot> object.
Run `rlang::last_trace()` to see where the error occurred.
```

Make sure you have the `patchwork` package loaded:

```
library(patchwork)
```

Issue 2: guide_train.prism_offset_minor error

You may encounter an error with `guide_train.prism_offset_minor`:

```
Error in guide_train.prism_offset_minor(guide, panel_params[[aesthetic]]) :
  No minor breaks exist, guide_prism_offset_minor needs minor breaks to work


Error in get(as.character(FUN), mode = "function"object envir = envir)
guide_prism_offset_minor' of mode'function' was not found
```

Ensure that the `ggprism` package is loaded:

```
library(ggprism)
```

Issue 3: SSL certificate problem

When encountering the following error:

```
SSL peer certificate or SSH remote key was not OK: [rest.kegg.jp] SSL certificate problem:
certificate has expired
```

If you are in China, make sure your computer network can bypass the firewall.

Issue 4: Bad Request (HTTP 400)

When encountering the following error:

```
Error in .getUrl(url, .flatFileParser) : Bad Request (HTTP 400).
```

Please restart R session.

Issue 5: Error in grid.Call(C_textBounds, as.graphicsAnnot(xlabel),xx, xx, xy, xy, :

When encountering the following error:

```
Error in grid.Call(C_textBounds, as.graphicsAnnot(xlabel),x$x, x$y, :
```

Please having some required fonts installed. You can refer to this thread.

Issue 6: Visualization becomes cluttered when there are more than 30 features of statistical significance.

When faced with this issue, consider the following solutions:

Solution 1: Utilize the 'select' parameter

The 'select' parameter allows you to specify which features you wish to visualize. Here's an example of how you can apply this in your code:

```
ggpicrust2::pathway_errorbar(
  abundance = kegg_abundance,
  daa_results_df = daa_results_df_annotated,
  Group = metadata$Day,
  p_values_threshold = 0.05,
  order = "pathway_class",
  select = c("ko05340", "ko00564", "ko00680", "ko00562", "ko03030", "ko00561", "ko00440",
"ko00250", "ko00740", "ko04940", "ko00010", "ko00195", "ko00760", "ko00920", "ko00311", "ko00310",
"ko04146", "ko00600", "ko04141", "ko04142", "ko00604", "ko04260", "ko00909", "ko04973", "ko00510",
"ko04974"),
  ko_to_kegg = TRUE,
  p_value_bar = FALSE,
  colors = NULL,
  x_lab = "pathway_name"
)
```

Solution 2: Limit to the Top 20 features

If there are too many significant features to visualize effectively, you might consider limiting your visualization to the top 20 features with the smallest adjusted p-values:

```
daa_results_df_annotated <-
daa_results_df_annotated[!is.na(daa_results_df_annotated$pathway_name),]

daa_results_df_annotated$p_adjust <- round(daa_results_df_annotated$p_adjust,5)

low_p_feature <- daa_results_df_annotated[order(daa_results_df_annotated$p_adjust),
]$feature[1:20]
```

```
p <- ggpicrust2::pathway_errorbar(
  abundance = kegg_abundance,
  daa_results_df = daa_results_df_annotated,
  Group = metadata$Day,
  p_values_threshold = 0.05,
  order = "pathway_class",
  select = low_p_feature,
  ko_to_kegg = TRUE,
  p_value_bar = FALSE,
  colors = NULL,
  x_lab = "pathway_name"
)
```

Issue 7: There are no statistically significant biomarkers

If you are not finding any statistically significant biomarkers in your analysis, there could be several reasons for this:

1. **The true difference between your groups is small or non-existent.** If the microbial communities or pathways you're comparing are truly similar, then it's correct and expected that you won't find significant differences.
2. **Your sample size might be too small to detect the differences.** Statistical power, the ability to detect differences if they exist, increases with sample size.
3. **The variation within your groups might be too large.** If there's a lot of variation in microbial communities within a single group, it can be hard to detect differences between groups.

Here are a few suggestions:

1. **Increase your sample size**: If possible, adding more samples to your analysis can increase your statistical power, making it easier to detect significant differences.
2. **Decrease intra-group variation**: If there's a lot of variation within your groups, consider whether there are outliers or subgroups that are driving this variation. You might need to clean your data, or to stratify your analysis to account for these subgroups.
3. **Change your statistical method or adjust parameters**: Depending on the nature of your data and your specific question, different statistical methods might be more or less powerful. If you're currently using a parametric test, consider using a non-parametric test, or vice versa. Also, consider whether adjusting the parameters of your current test might help.

Remember, not finding significant results is also a result and can be informative, as it might indicate that there are no substantial differences between the groups you're studying. It's important to interpret your results in the context of your specific study and not to force statistical significance where there isn't any.

With these strategies, you should be able to create a more readable and informative visualization, even when dealing with a large number of significant features.
