# Supplementary material for: ggpicrust2: an R package for PICRUSt2 predicted functional profile analysis and visualization
Source: Bioinformatics. 2023 Aug 1;39(8):btad470. doi: 10.1093/bioinformatics/btad470 (PMC10425198; doi:10.1093/bioinformatics/btad470)
Supplement: btad470_Supplementary_Data [file btad470_supplementary_data.zip › Supplementary_materials 1.docx]

*Supplementary materials for: ggpicrust2*: an R package for PICRUSt2 pre-dicted functional profile analysis and visualization

## Supplementary Table S1: Summary table of input data type, advantages and limitations of differential abundance methods implemented in ggpicrust2 package

| Method | Input data | Pros | Cons |
| --- | --- | --- | --- |
| LinDA | Count or abundance data | 1. Simple, robust and highly scalable approach to tackle the compositional effects; 2. Only requires fitting linear regression models on the centered log-ratio transformed data and correcting the bias; 3. Enjoys asymptotic FDR control and can be extended to mixed-effect models for correlated microbiome data | 1. May not be suitable for zero-inflated data or non-linear relationships |
| ALDEx2 | Count data | 1. Suitable for rare features; 2. Variance stabilizing transformation; 2. Good for comparing vastly different microbiome samples | 1. Greatly affected by sample size; 2. May undermine downstream analyses for subtle effect sizes |
| DESeq2 | Count data | 1. Size factor normalization; 2. Models counts with the Negative Binomial; 2. Can handle more complex tests and experimental designs | 1. Based on negative binomial model, requiring similar library sizes across samples; 2. May not be the best choice for certain special conditions, such as a large proportion of true positives and sufficient replicates, small sample sizes, or extreme values |
| edgeR | Count data | 1. Models counts with the Negative Binomial; 2. Can accurately and specifically detect differential abundance over the full range of effect sizes, replicate numbers, and library sizes | 1. Requires high sequencing depth for detecting differential abundance; 2. May have increased Type-I error compared to later methods |
| limma | Count or abundance data | 1. Easy to use; 2. Can be used with voom to detect low abundance differences | 1. Cannot adjust for differences in sequencing depth by itself |
| metagenomeSeq | Abundance data | 1. No need to assume the distribution of the remaining zeros; 2. Considers sequencing depth differences; 3. Uses a zero-inflated Gaussian mixture model | 1. Slower; 2. Results may be affected by sequencing depth; 3. May not be the best choice when biological replicate samples are low; may have a higher rate of false positives |
